# Enhanced insulin receptor, but not PI3K, signalling protects podocytes from ER stress

**DOI:** 10.1038/s41598-018-22233-9

**Published:** 2018-03-02

**Authors:** Kathryn L. Garner, Virginie M. S. Betin, Vanda Pinto, Mark Graham, Emmanuelle Abgueguen, Matt Barnes, David C. Bedford, Craig A. McArdle, Richard J. M. Coward

**Affiliations:** 1 Bristol Renal, Bristol Medical School, University of Bristol, Dorothy Hodgkin Building, Whitson Street, Bristol, BS1 3NY UK; 2 Takeda Cambridge Ltd., 418 Cambridge Science Park, Milton Road, Cambridge, CB4 0PZ UK; 3 Laboratories for Integrative Neuroscience and Endocrinology, Bristol Medical School, University of Bristol, Dorothy Hodgkin Building, Whitson Street, Bristol, BS1 3NY UK

## Abstract

Disruption of the insulin-PI3K-Akt signalling pathway in kidney podocytes causes endoplasmic reticulum (ER) stress, leading to podocyte apoptosis and proteinuria in diabetic nephropathy. We hypothesised that by improving insulin sensitivity we could protect podocytes from ER stress. Here we use established activating transcription factor 6 (ATF6)- and ER stress element (ERSE)-luciferase assays alongside a novel high throughput imaging-based C/EBP homologous protein (CHOP) assay to examine three models of improved insulin sensitivity. We find that by improving insulin sensitivity at the level of the insulin receptor (IR), either by IR over-expression or by knocking down the negative regulator of IR activity, protein tyrosine-phosphatase 1B (PTP1B), podocytes are protected from ER stress caused by fatty acids or diabetic media containing high glucose, high insulin and inflammatory cytokines TNFα and IL-6. However, contrary to this, knockdown of the negative regulator of PI3K-Akt signalling, phosphatase and tensin homolog deleted from chromosome 10 (PTEN), sensitizes podocytes to ER stress and apoptosis, despite increasing Akt phosphorylation. This indicates that protection from ER stress is conferred through not just the PI3K-Akt pathway, and indeed we find that inhibiting the MEK/ERK signalling pathway rescues PTEN knockdown podocytes from ER stress.

## Introduction

Diabetic nephropathy (DN) is the leading global cause of end-stage renal disease^1,2^, accounting for nearly 50% of patients in the United States requiring dialysis or a kidney transplant^3^. The natural history of DN is dominated by progressive albuminuria due to damage of the glomerular filtration barrier (GFB)^4,5^. Podocytes are the major constituent cell of the kidney glomerulus; with their long finger-like projections they form the specialised filter through which waste products pass from the blood into the urine. In DN, podocyte injury leads to GFB disruption resulting in proteinuria and further kidney damage. Under these conditions podocytes develop insulin resistance, foot process effacement, cell detachment and apoptosis, leading to increased GFB permeability^6–8^.

In DN, hyperglycaemia^9^, free fatty acids^10^ and defective insulin signalling^11^ can lead to the development of endoplasmic reticulum (ER) stress in podocytes^12^. Under normal physiological conditions, newly synthesised proteins must be properly folded in the ER so that they meet cellular quality control criteria for ER exit. Where protein synthesis is high or folding is impaired, the protein folding machinery can become overwhelmed, triggering a series of adaptive responses known as the unfolded protein response (UPR). Initially, the UPR serves to increase the fidelity and/or efficiency of protein folding. For example, liberation of the transcription factor ATF6 from the ER membrane leads to ATF6-driven upregulation of the ER-specific protein chaperone BiP/GRP78 to help misfolded proteins fold properly. Such increases in chaperone expression are co-ordinated with inhibition of protein translation and activation of ER-associated degradation (ERAD) that directs misfolded proteins for degradation. For example, Herp (encoded by the *HERPUD1* gene; homocysteine-inducible, endoplasmic reticulum stress-inducible, ubiquitin-like domain member 1) is a member of the ERAD pathway, and by interacting with components of the ubiquitin family it helps to target irrevocably misfolded proteins to the proteasome for degradation^13^. However, if these adaptive responses are unable to bring this ER stress under control and reverse it within a certain timeframe, apoptosis is initiated to eliminate the damaged cells. Among multiple mechanisms, binding of ATF6 to an ER stress response element (ERSE)^14^ drives the expression of the pro-apoptotic transcription factor C/EBP homologous protein (CHOP), which interacts with Bcl-2 family members to increase mitochondrial cell death signals^15–18^.

Podocytes are insulin-responsive cells^8,19,20^, and since defective insulin signalling is associated with the development of ER stress^11^, we hypothesised that improving insulin sensitivity would protect podocytes from this stress. We developed and applied transcriptional and imaging readouts for ER stress in podocytes in order to interrogate different aspects of the UPR in podocyte cell lines genetically modified to improve insulin sensitivity. We show that known chemical activators of ER stress increase ATF6- and ERSE-luciferase activity in podocytes, and also increase the nuclear expression of CHOP, as measured by high content imaging of immunostained cells. Insulin sensitivity was increased by insulin receptor (IR) over-expression, protein tyrosine-phosphatase 1B (PTP1B) knockdown, and phosphatase and tensin homolog deleted from chromosome 10 (PTEN) knockdown, confirmed by measuring the insulin-stimulated phosphorylation of Akt. Consistent with our working hypothesis, improving insulin sensitivity by IR overexpression or by PTP1B knockdown protected podocytes from ER stress. However, surprisingly, the increase in insulin sensitivity caused by PTEN knockdown was actually associated with an increase in ER stress and apoptosis, and this stress could be suppressed by inhibitors of MEK and ERK. We conclude that the relationship between insulin sensitivity and ER stress is more complex than anticipated; the protection from ER stress conferred by improving insulin sensitivity is clearly not communicated through the PI3K-Akt pathway alone.

## Results

### Thapsigargin, tunicamycin and free fatty acids activate ER stress in podocytes

**Figure 1:**
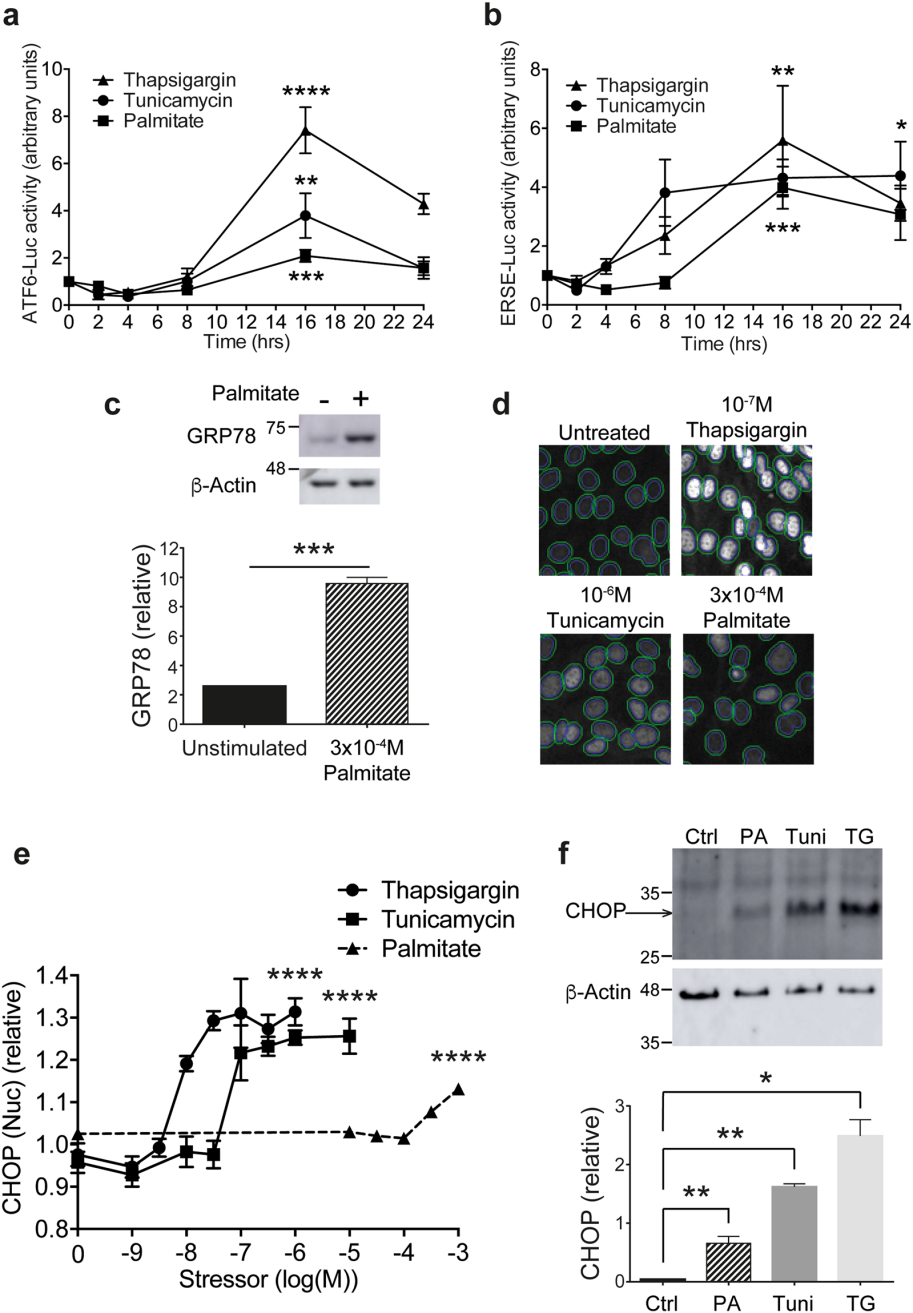
Stimulation of ER stress in podocytes. (**a**) ATF6-luciferase activity monitored after treatment with 2, 4, 8, 16 or 24 hr 100 nM thapsigargin, 1 µM tunicamycin or 300 µM palmitate (*n* = 4). One-way ANOVA, thapsigargin ****p < 0.0001, tunicamycin **p = 0.0012, palmitate ****p = 0.0009. A Tukey’s *post-hoc* comparison comparing each timepoint to 0 hr revealed statistical significance at 16 hr (****) and 24 hr (**) for thapsigargin treatment, at 16 hr (**) for tunicamycin, and at 16 hr (*) for palmitate. (**b**) ERSE-luciferase activity monitored after treatment with 2, 4, 8, 16 or 24 hr 100 nM thapsigargin, 1 µM tunicamycin or 300 µM palmitate (*n* = 4). One-way ANOVA thapsigargin *p = 0.0122, tunicamycin **p = 0.0043, palmitate ***p = 0.0001. A Tukey’s *post-hoc* comparison comparing each timepoint to 0 hr revealed statistical significance at 16 hr (*) thapsigargin treatment, and at 16 hr (**) for palmitate. (**c**) Representative western blot of the increase in GRP78 expression observed when podocytes were treated with 300 µM palmitate. Densitometric quantification normalised to β-actin (*n* = 3). Paired *t* test, ***p < 0.001. (Full blot shown in Supplementary Fig. 3). (**d**) Representative images of the CHOP immunoassay acquired with the IN Cell Analyzer. DAPI staining in the blue channel was used to segment the nuclei and was then mapped onto the green channel (CHOP immunofluorescence), shown by the inner line, and CHOP fluorescence intensity in the nucleus quantified. (**e**) CHOP nuclear intensity in podocytes relative to vehicle controls exposed to 10^−9^-10^−6^ M Thapsigargin, 10^−9^-10^−5^ M Tunicamycin or 10^−5^-10^−3^ M Palmitate (*n* = 3). Log EC_50_ values were −8.14 for Thapsigargin, −7.22 for Tunicamycin and −2.73 for Palmitate. AFU, arbitrary fluorescence units. One-way ANOVA, thapsigargin ****p < 0.0001, tunicamycin ****p < 0.0001, palmitate ****p < 0.0001. A Tukey’s *post-hoc* comparison comparing each stressor concentration to unstimulated controls revealed statistical significance at 10^−8^ M (**) and 10^−7.5^-10^−6^ M (****) for thapsigargin, 10^−7^-10^−6.5^ M (***) and 10^−6^-10^−5^ M (****) for tunicamycin, and 10^−3.5^ M (**) and 10^−3^ M (****) for palmitate. (**f**) Representative western blot for CHOP with densitometric quantification (*n* = 3), using the same antibody as used for immunofluorescence in (**e**). WT podocytes untreated or treated for 24 hr with 100 nM thapsigargin (‘TG’), 1 µM tunicamycin (‘Tuni’), or 300 µM palmitate (‘PA’). Paired *t* test, *p < 0.05, **p < 0.01.

A key consequence of the UPR is the upregulation of ER-specific chaperone proteins to help fold misfolded proteins. In order to measure ER stress responses in immortalised mouse podocytes, we began by using two luciferase assays to monitor ATF6- and ERSE-driven gene transcription. Stable podocyte cell lines were made through transduction of lentiviruses to stably express the ER stress-specific promoter sequences ATF6 or ERSE driving the firefly luciferase reporter, together with renilla luciferase expressed independently as an internal control. We tested these assays using thapsigargin and tunicamycin, traditionally used as chemical inducers of ER stress. Thapsigargin blocks sarco/endoplasmic reticulum Ca^2+^ATPase (SERCA) to prevent the pumping of Ca^2+^ ions back into the ER, whereas tunicamycin prevents *N*-linked protein glycosylation. Both thapsigargin and tunicamycin induced time-dependent increases in ATF6-driven (Fig. 1a) and ERSE-driven (Fig. 1b) luciferase activity. Peak activity was observed at 16 hr; after this time luciferase activity declined, likely due to a reduction in luciferase protein as induction of the UPR halts general protein translation. Dose response experiments to thapsigargin and tunicamycin were carried out alongside the time response experiments; log EC_50_ values for concentration for ATF6-Luc were −7.66 for thapsigargin and −7.40 for tunicamycin, while for ERSE-Luc log EC_50_ values were −8.95 for thapsigargin and −8.03 for tunicamycin.

Palmitate, one of the most abundant free fatty acids in blood plasma^21^, has been found in higher quantities in the blood of patients with type 2 diabetes^22^, and has been shown to induce insulin resistance in immortalized human podocytes^23^. Furthermore, palmitate has previously been shown to induce ER stress and cell death in podocytes^24^. This endogenous ER stressor induced time-dependent increases in ATF6-driven (Fig. 1a) and ERSE-driven (Fig. 1b) luciferase activity, with maximum responses achieved at 16 hr treatment. For palmitate concentration, the log EC_50_ values were −3.91 for ATF6-Luc and −4.55 for ERSE-Luc. Monitoring GRP78 expression, a downstream target of ATF6 and ERSE, in podocytes treated with 300 μM palmitate by western blotting, confirmed the increase in luciferase activity we observed (Fig. 1c).

In addition to monitoring gene transcription using commercially-available luciferase assays, we developed a high content imaging-based assay. This enabled us to monitor the upregulation of ER stress-inducible proteins downstream of the increase in transcription, and to do so in single cells rather than in the whole cell population. All three ER stressors induced a robust dose-dependent upregulation of CHOP expression in the nucleus of podocytes after 24 hr (Fig. 1d,e). At this timepoint, although ER stress was induced, no cell loss was observed; at 48 hr and beyond, a notable loss of cells was observed as apoptosis was initiated (Supplementary Fig. 1). Western blotting confirmed that total cellular CHOP expression was significantly increased in podocytes in response to all three stressors after 24 hr (Fig. 1f).

### Insulin receptor over-expression protects podocytes from ER stress and apoptosis

**Figure 2:**
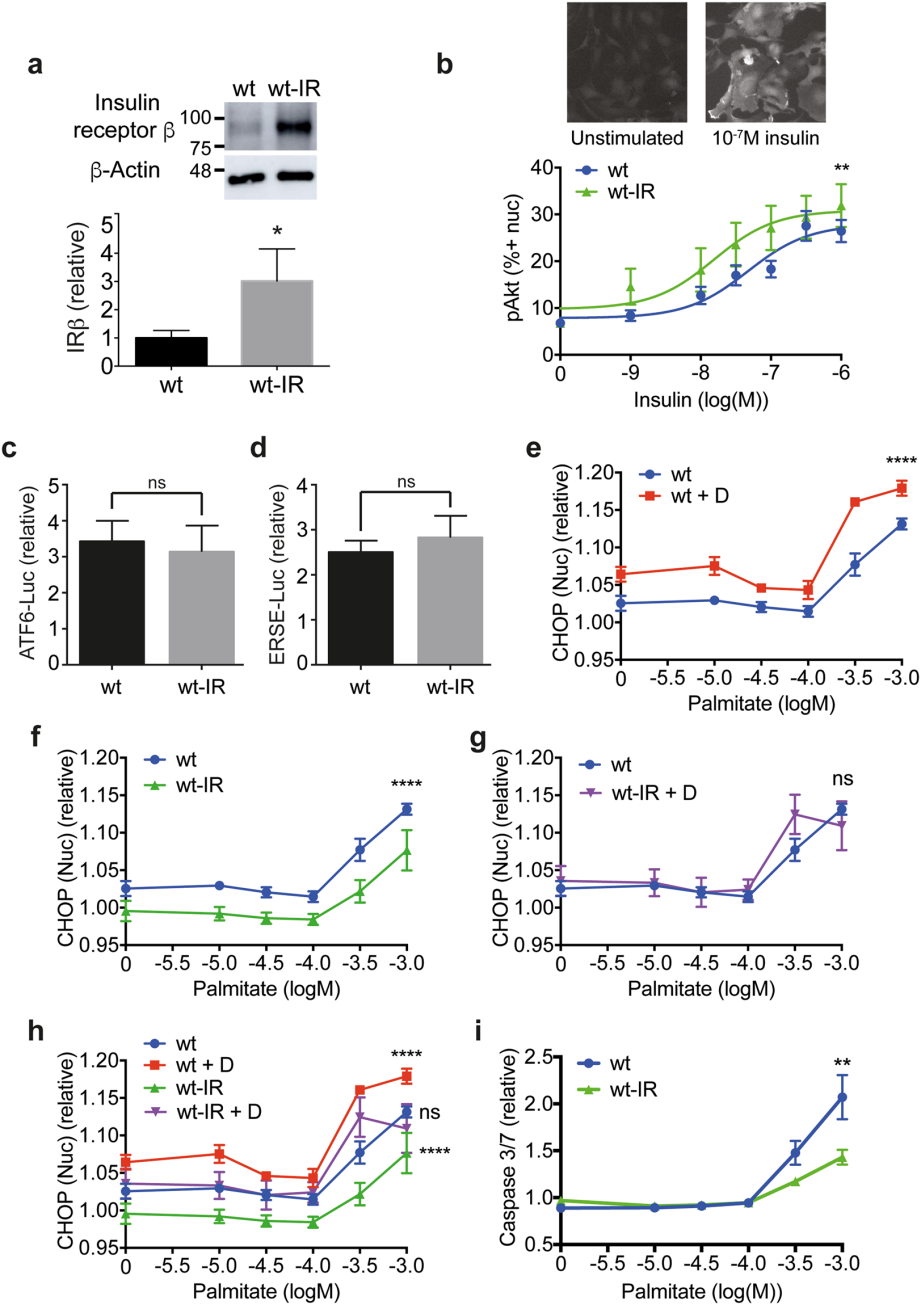
IR over-expression protects against ER stress. (**a**) Representative western blot with densitometric quantification normalised to β-actin (*n* = 3) showing IRβ subunit expression is increased 3-fold in wt-IR podocytes compared with wt cells. Unpaired *t* test, *p = 0.0153. (Full blot shown in Supplementary Fig. 3). (**b**) Insulin sensitivity monitored by pAkt immunostaining in podocytes following 10^−9^-10^−6^ M insulin stimulation with representative images for wt-IR podocytes unstimulated or stimulated with 10^−7^ M insulin for 10 min. 10 and 15 minute timepoints were combined (*n* = 8), and the data expressed as the percentage of cells positive for pAkt in the nucleus. Two-way ANOVA, effect of IR over-expression **p = 0.0057, effect of insulin concentration ****p < 0.0001, with no significant interaction between the effect of IR over-expression and insulin concentration. A Bonferroni *post-hoc* comparison revealed no significant difference between wt and wt-IR cells at each insulin concentration. (**c**) ATF6-driven luciferase activity for wt and wt-IR podocytes treated with diabetic media relative to normal growth media. Unpaired *t* test with Welch’s correction (*n* = 6), not significant, ‘ns’. (**d**) ERSE-driven luciferase activity for wt and wt-IR podocytes treated with diabetic media relative to normal growth media. Unpaired *t* test with Welch’s correction (*n* = 6), not significant, ‘ns’. (**e**) CHOP response to 10^−5^-10^−3^ M palmitate for podocytes differentiated in normal growth media or diabetic media (‘D’), (*n* = 3). Two-way ANOVA, effect of palmitate concentration ****p < 0.0001, effect of diabetic media ****p < 0.0001, with a significant interaction between palmitate concentration and diabetic media *p = 0.0330. A Bonferroni *post-hoc* comparison comparing podocytes differentiated in normal growth media or diabetic media revealed statistical significance at 0 M (*), 10^−5.5^ M and 10^−3^ M (**), and 10^−3.5^ M (****) palmitate. (**f**) CHOP response to 10^−5^-10^−3^ M Palmitate, wt podocytes compared to wt-IR, (*n* = 3). Two-way ANOVA, effect of IR over-expression ****p < 0.0001, effect of palmitate concentration ****p < 0.0001, with no significant interaction between the effect of IR over-expression and palmitate concentration. A Bonferroni *post-hoc* comparison comparing wt and wt-IR podocytes revealed statistical significance at 10^−3.5^-10^−3^ M (*) palmitate. (**g**) CHOP response to palmitate for wt podocytes compared with wt-IR treated with diabetic media, (*n* = 3). Two-way ANOVA, effect of palmitate concentration ****p < 0.0001, but no significant effect of wt compared with wt-IR + D. (**h**) Composite graph of curves shown in (**e**,**f**) and (**g**). (**i**) Caspase 3/7 activation as a measure of apoptosis in wt and wt-IR podocytes following 24 hr palmitate treatment (*n* = 3). Two-way ANOVA, effect of IR over-expression **p = 0.0044, effect of palmitate ****p < 0.0001, with a significant interaction between IR over-expression and palmitate dose ****p < 0.0001. A Bonferroni *post-hoc* comparison comparing wt and wt-IR revealed statistical significance at 10^−3^ M palmitate (****).

Podocytes are insulin-responsive cells, and mice with podocyte-specific IR knockdown develop significant albuminuria^8^. Disruption of the PI 3-kinase (PI3K)-Akt signalling pathway through genetic ablation of the IR or PI3K subunits causes ER stress^11^, and we therefore hypothesised that improving insulin sensitivity would protect podocytes from ER stress. We began by examining a podocyte cell line with stable over-expression of IR^25^. Expression of the IR in these wild-type (wt)-IR cells was found to be three-fold higher than in wt podocytes by western blot (Fig. 2a). Concomitant with the increase in IR expression, wt-IR were more sensitive to insulin, as shown recently^25^, and we demonstrate this here using high content immunofluorescence imaging of insulin-induced phosphorylation of Akt at Ser473 (Fig. 2b).

We recently developed conditions for culturing podocytes which mimic those experienced by podocytes during diabetes, an environment rich in glucose, insulin and inflammatory cytokines, TNFα and IL-6, which promotes insulin resistance^25^. We observed that incubating podocytes with diabetic media during differentiation increased both ATF6- and ERSE-luciferase activity, but this was unaffected by IR over-expression (Fig. 2c,d).

Similarly, diabetic media significantly increased nuclear CHOP expression, and this acted synergistically to upregulate CHOP induced by a range of palmitate concentrations (Fig. 2e,h). Conversely, CHOP induction was significantly attenuated in podocytes over-expressing the IR, in both untreated and palmitate-treated cells (Fig. 2f,h). Notably, treatment of wt-IR podocytes with a range of palmitate concentrations on a background of diabetic media displayed CHOP induction indistinguishable from wt treated in the absence of diabetic media (Fig. 2g,h), indicating the ability of IR over-expression to protect podocytes from ER stress. This protection from ER stress translated into protection from apoptosis (Fig. 2i).

We additionally assessed markers of ER stress by western blotting. Induction of GRP78 expression and phosphorylation of PERK in response to palmitate treatment on a background of diabetic media were completely suppressed by IR over-expression (Supplementary Fig. 2), again supporting our finding that IR over-expression protects podocytes from the development of ER stress.

### PTP1B knockdown protects against ER stress

**Figure 3:**
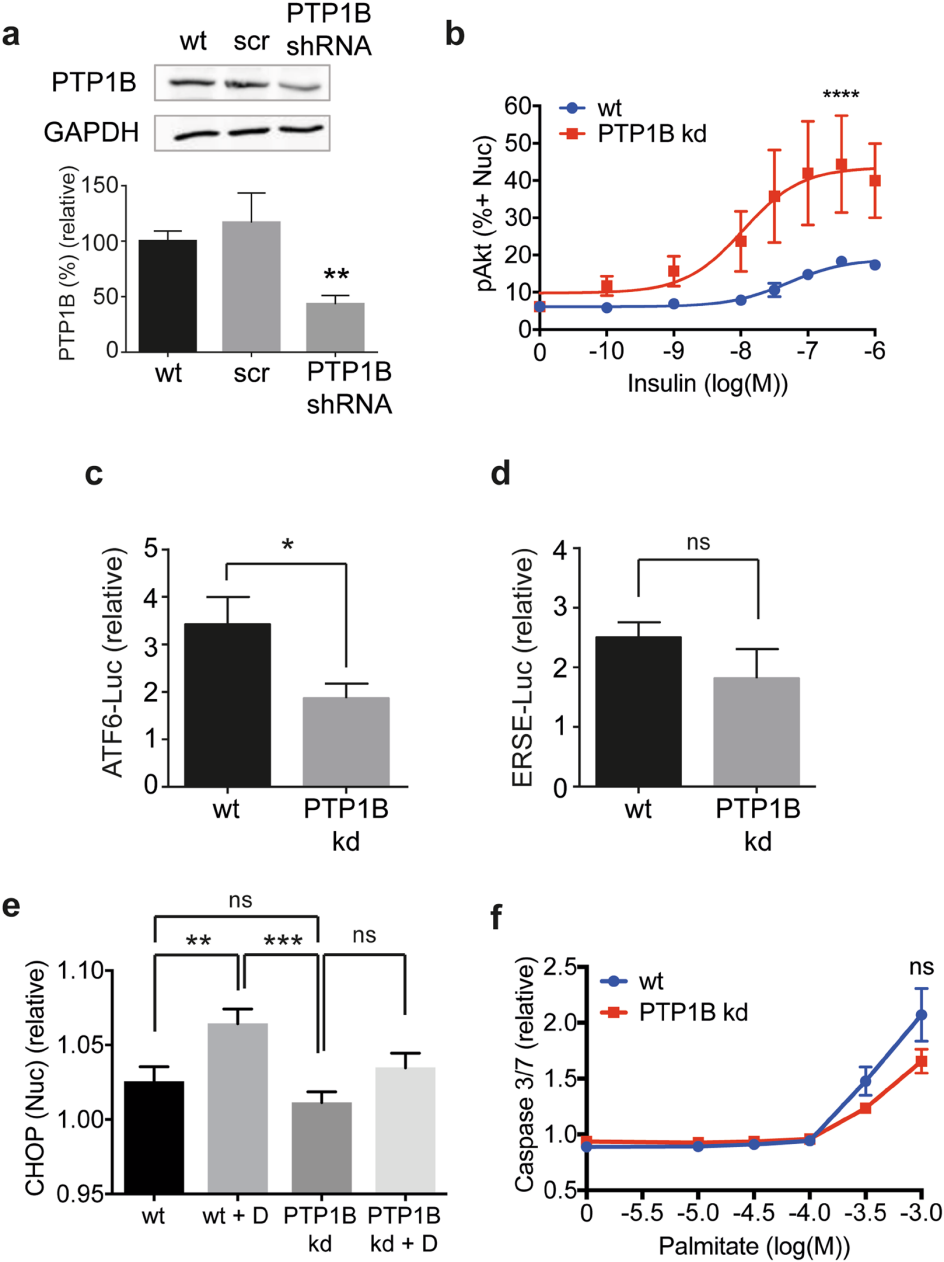
Knockdown of PTP1B protects against ER stress. (**a**) Representative western blot with densitometric quantification normalised to GAPDH demonstrating PTP1B knockdown of 56% in the PTP1B kd cell line compared with wt cells (*n* = 3). Unpaired *t* test **p = 0.005. Quantification of PTP1B in cells treated with scrambled (scr) shRNA also shown. (Full blot shown in Supplementary Fig. 3). (**b**) Insulin sensitivity monitored by pAkt immunostaining in cell nuclei following 10 min of 10^−10^-10^−6^ M insulin stimulation (*n* = 4). Data expressed as the percentage of cells positive for pAkt in the nucleus. Two-way ANOVA, effect of PTP1B *p* < 0.0001****, insulin ***p = 0.0002, but no significant interaction between the effect of PTP1B and insulin concentration. A Bonferroni *post-hoc* comparison revealed statistical significance at 10^−7^ M insulin (*). (**c**) ATF6-driven luciferase activity for wt and PTP1B kd podocytes treated with diabetic media relative to normal growth media. Unpaired *t* test with Welch’s correction (*n* = 3), *p < 0.05. (**d**) ERSE-driven luciferase activity for wt and PTP1B kd podocytes treated with diabetic media relative to normal growth media. Unpaired *t* test with Welch’s correction (*n* = 3). (**e**) CHOP response in wt and PTP1B kd podocytes for cells treated with or without diabetic media (‘D’), (*n* = 3). Comparison of wt and PTP1B kd, unpaired *t* test with Welch’s correction; paired *t* test for comparison of wt and wt + D *p* = 0.0091 (**), wt + D and PTP1B kd *p* = 0.0006 (***), PTP1B kd and PTP1B kd + D (ns). (**f**) Caspase 3/7 activation in wt and PTP1B kd podocytes following 24 hr palmitate treatment (*n* = 3). Two-way ANOVA, effect of PTP1B kd not significant, effect of palmitate ****p < 0.0001.

PTP1B acts as a negative regulator of the insulin signalling pathway by dephosphorylating the IR and IR substrate 1 (IRS1)^26^. We created a PTP1B knockdown cell line by transducing immortalised podocytes with a lentivirus to stably express PTP1B shRNA. Knockdown of PTP1B by 56% was achieved (Fig. 3a), yet insulin sensitivity as measured by phosphorylation of Akt was significantly increased (Fig. 3b).

Furthermore, in podocytes incubated with diabetic media, ATF6-driven luciferase activity was significantly reduced in PTP1B knockdown (kd) cells relative to wild-type cells (Fig. 3c), indicating that PTP1B knockdown can protect podocytes from the development of ER stress. A similar trend was seen for ERSE-driven luciferase activity, but this was not found to be significant (Fig. 3d).

Although diabetic media upregulated CHOP expression in wt podocytes, interestingly the same CHOP induction was not observed in PTP1B kd podocytes, and there was no significant difference between untreated wt and PTP1B kd cells with regards to CHOP expression in the nucleus (Fig. 3e). This again indicates that PTP1B knockdown protects podocytes from ER stress. We observed a trend for PTP1B knockdown to suppress apoptosis in podocytes treated with a range of palmitate concentrations, but this was not found to be significant (Fig. 3f).

**Figure 4:**
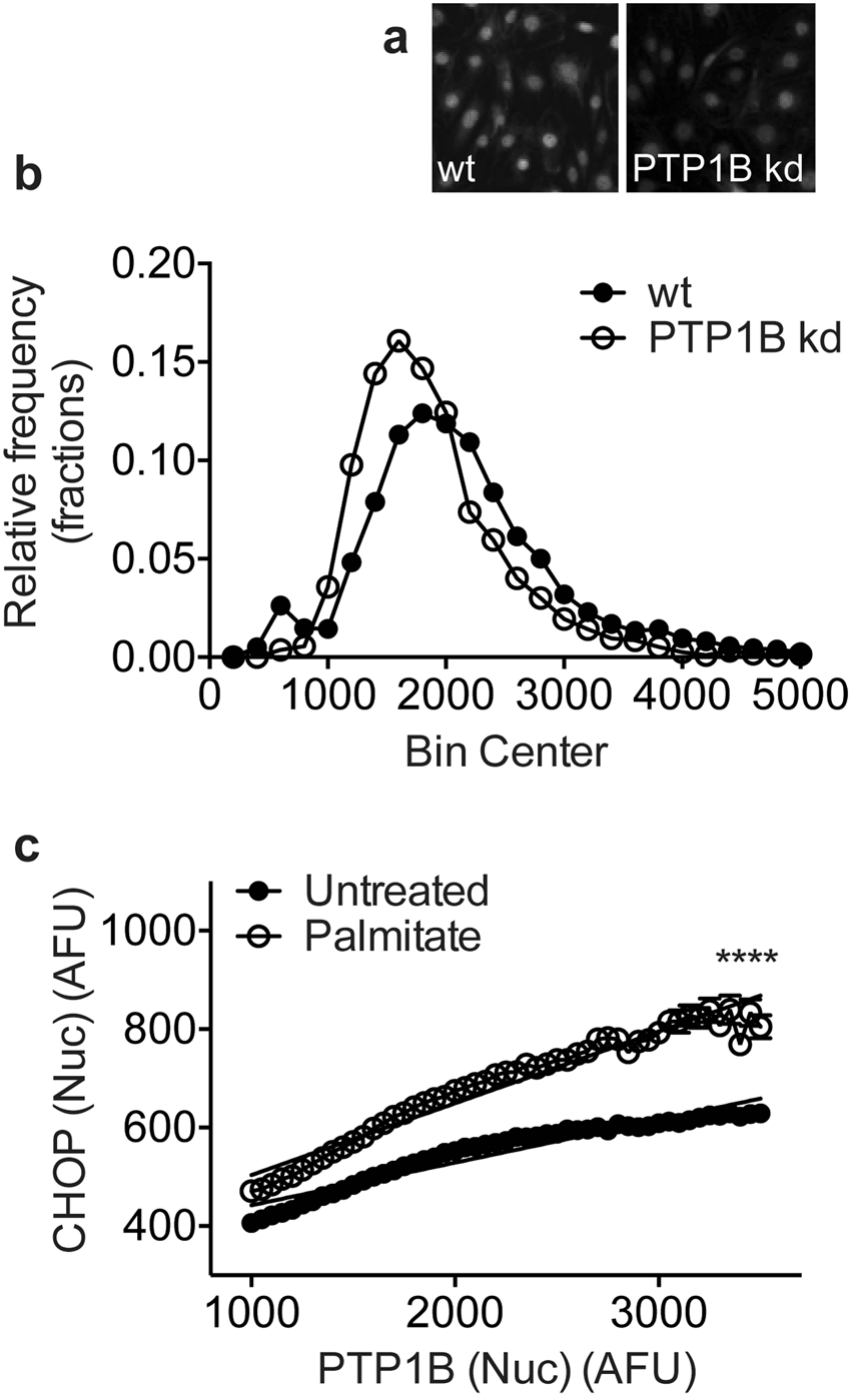
The amount of PTP1B in podocytes positively correlates with ER stress. WT and PTP1B kd cells untreated or treated with 300 µM palmitate were stained for CHOP, PTP1B and with DAPI. (**a**) Immunofluorescent staining of wt and PTP1B kd cells for PTP1B. (**b**) Frequency distribution plot of unadjusted PTP1B intensity values for single cells sorted into 50-AFU bins. (**c**) Nuclear PTP1B and CHOP paired single cell values were sorted based on the amount of PTP1B into 50-AFU bins. WT and PTP1B kd cells were combined *in silico* to yield a wide range of PTP1B intensity values for cells untreated or palmitate-treated. Within each bin, the mean CHOP value was calculated and plotted. Linear regression analysis showed that both slopes were significantly non-zero, *p* < 0.0001. R^2^ = 0.92 for untreated podocytes and 0.95 for palmitate-treated. Two-way ANOVA, effect of PTP1B expression ****p < 0.0001, effect of palmitate treatment ****p < 0.0001, with a significant interaction between PTP1B expression and palmitate treatment ****p < 0.0001. Bonferroni *post-hoc* comparison comparing row means (CHOP expression without or with palmitate treatment for each value of PTP1B) revealed means were significantly different (****p < 0.0001) for each value of PTP1B.

Since the reduction in PTP1B expression in PTP1B kd cells was moderate, we sought to demonstrate the relationship between PTP1B expression and CHOP induction in single cells by co-staining podocytes untreated or treated with 300 µM palmitate with antibodies recognising PTP1B and CHOP (Fig. 1a). The frequency distribution plot in Fig. 4b demonstrates the range of nuclear PTP1B intensity values observed for single cells in a population of wt compared with those in a population of PTP1B kd cells. Cells were sorted *in silico* into 50-AFU bins based on the amount of PTP1B expressed, and mean CHOP expression for cells within each bin was determined. A positive correlation was observed between PTP1B and CHOP expression, which increased when cells were treated with palmitate (Fig. 4c). This indicates that the degree of CHOP upregulation in a single cell is proportional to the amount of PTP1B present, and that this effect is increased when cells are treated with palmitate.

### PTEN knockdown surprisingly increases ER stress

**Figure 5:**
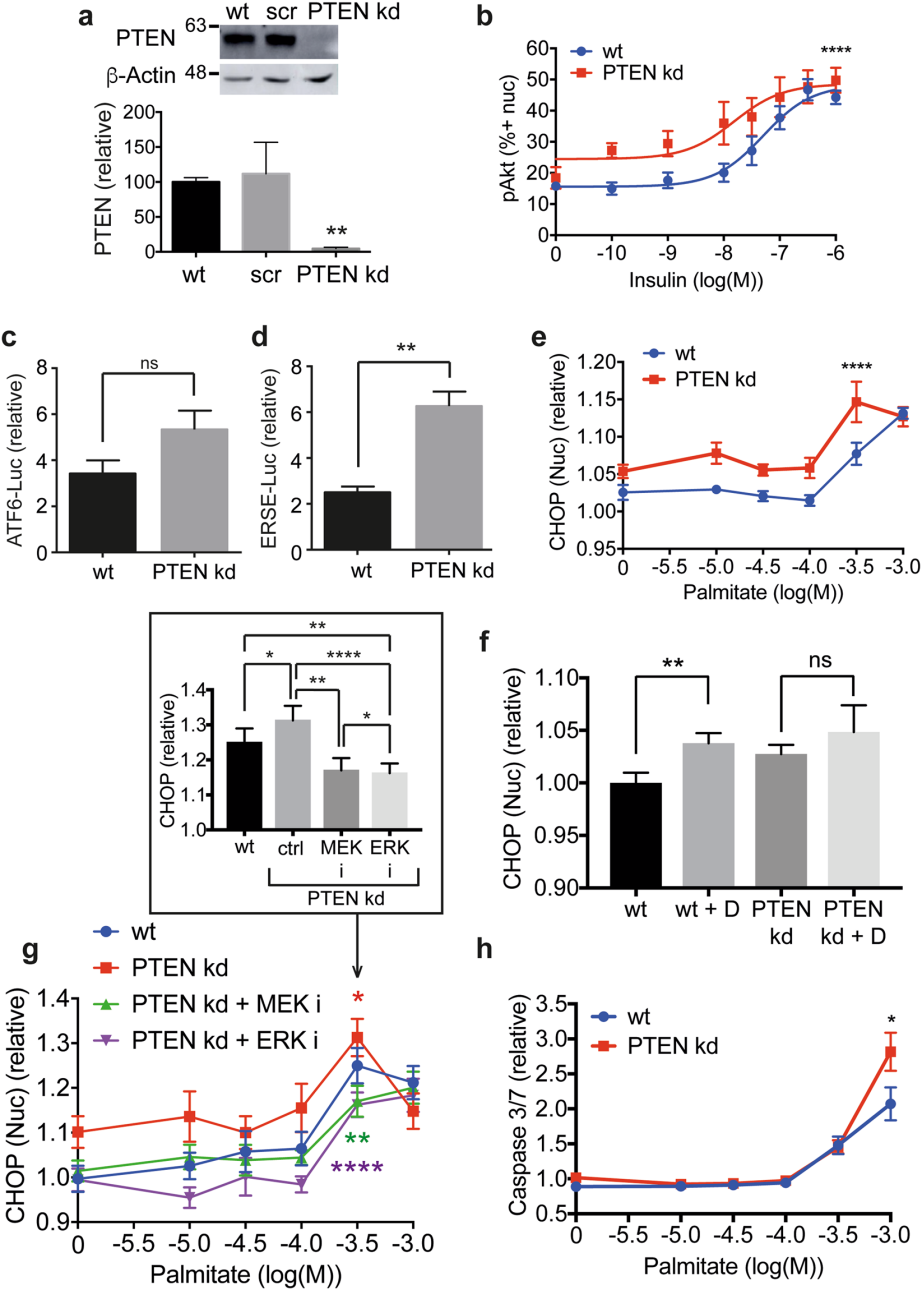
Knockdown of PTEN in podocytes increases ER stress. (**a**) Representative western blot with densitometric quantification normalised to β-actin demonstrating near-complete knockdown of PTEN using shRNA compared with wt cells and cells treated with scrambled (scr) shRNA, (*n* = 3). Unpaired *t* test with Welch’s correction (wt vs PTEN shRNA **p = 0.0022). (Full blot shown in Supplementary Fig. 3). (**b**) Insulin sensitivity monitored by pAkt immunostaining in cell nuclei following 10 minutes of 10^−10^-10^−6^ M insulin stimulation (*n* = 4). Data expressed as the percentage of cells positive for pAkt in the nucleus. Two-way ANOVA, effect of PTEN *p* < 0.0001****, insulin ****p < 0.0001, but no significant interaction between the effect of PTEN knockdown and insulin concentration. A Bonferroni *post-hoc* comparison revealed no significant difference between wt and PTEN kd cells at each insulin concentration. (**c**) ATF6-driven luciferase activity for wt and PTEN kd podocytes treated with diabetic media relative to normal growth media. Unpaired *t* test with Welch’s correction (*n* = 3), not significant, ‘ns’. (**d**) ERSE-driven luciferase activity for wt and PTEN kd podocytes treated with diabetic media relative to normal growth media. Unpaired *t* test with Welch’s correction (*n* = 3), **p < 0.01 (*n* = 3). (**e**) WT and PTEN kd podocytes treated with 10^−5^-10^−3^ M Palmitate for 24 hr were fixed and stained with DAPI and for CHOP (*n* = 3). Two-way ANOVA *p* < 0.0001 for cell type (****) and for palmitate concentration (****), with no significant interaction between cell type and palmitate concentration. A Bonferroni *post-hoc* comparison revealed statistical significance at 10^−5^ M (*) and 10^−3.5^ M (**) palmitate. (**f**) WT and PTEN kd cells were differentiated in the presence of either normal growth media or diabetic (‘D’) media for 14 days before being fixed and immunostained for CHOP and with DAPI (*n* = 3). Paired *t* test: wt and wt + D, *p* = 0.0091 (**); PTEN kd and PTEN kd + D, *p* = 0.4737 (ns). (**g**) PTEN kd podocytes treated with 10^−5^-10^−3^ M Palmitate were additionally treated with 10 µM MEK (PD 184352) and 30 µM ERK (FR 180204) inhibitors (‘**i**’) for 24 hr. Two-way ANOVA, wt vs PTEN kd *p = 0.0166; PTEN kd vs PTEN kd + MEK inhibitor ** *p* = 0.0012; PTEN kd vs PTEN kd + ERK inhibitor ****p < 0.0001; wt vs PTEN kd + MEK inhibitor not significant; wt vs PTEN kd + ERK inhibitor **p = 0.0065; PTEN kd + MEK inhibitor vs + ERK inhibitor *p = 0.0264. Effect of palmitate ****p < 0.0001 in all comparisons. A Bonferroni *post-hoc* comparison for PTEN kd cells -/ + ERK inhibitor revealed statistical significance at 10^−5^ M (**) and 10^−4^-10^−3.5^ M palmitate (*). *Inset* Bar graph of 10^3.5^ M palmitate values with two-way ANOVA statistics from whole palmitate ranges shown. (**h**) Caspase 3/7 activation in wt and PTEN kd podocytes following 24 hr palmitate treatment (*n* = 3). Two-way ANOVA, effect of PTEN kd *p = 0.0208, effect of palmitate ****p < 0.0001, with a significant interaction between PTEN kd and palmitate dose **p = 0.009. A Bonferroni *post-hoc* comparison for wt and PTEN kd revealed statistical significance at 10^−3^ M palmitate (****).

Our final strategy to improve podocyte insulin sensitivity was to make a cell line with stable knockdown of PTEN using shRNA. Insulin activates the PI3K-Akt and Raf-MEK-ERK signalling pathways in podocytes^8^, and by dephosphorylating the 3′-position of the inositol ring of PI(3,4,5)P_3_, PTEN acts as a negative regulator of the PI3K-Akt pathway downstream of insulin. PTEN expression was reduced by 95% in this cell line (Fig. 5a). PTEN kd podocytes exhibited a significantly increased sensitivity to insulin, shown by increased Akt phosphorylation (Fig. 5b).

Unexpectedly, however, ER stress induction was increased in PTEN kd cells. Although the observed increase in ATF6-driven luciferase activity in PTEN kd compared to wt was not significant (Fig. 5c), that seen for the ERSE-luciferase reporter was found to be significant (Fig. 5d), and highly significant for CHOP upregulation (Fig. 5e). Importantly, there was no significant increase in CHOP expression for PTEN kd podocytes treated with diabetic media compared with untreated PTEN kd podocytes (Fig. 5f). To investigate this further we treated PTEN kd cells with MEK and ERK inhibitors along with a range of palmitate concentrations, and found that inhibiting the ERK signalling pathway resulted in a significant reduction in nuclear CHOP in PTEN-depleted podocytes (Fig. 5g). Downstream of ER stress, we observed that caspase 3/7 activation was significantly increased in PTEN kd cells treated with palmitate relative to wt podocytes, indicating an increased rate of apoptosis in these cells (Fig. 5h).

## Discussion

**Figure 6:**
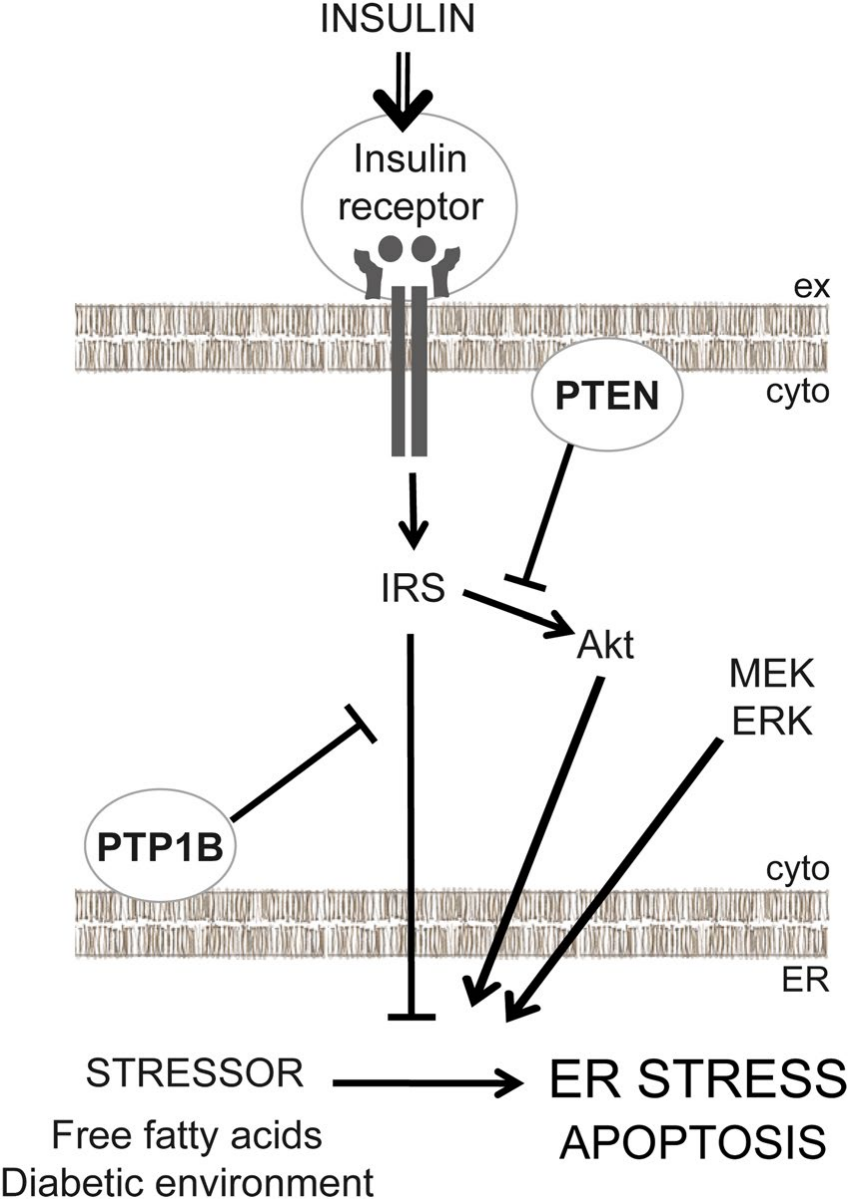
Summary. When insulin sensitivity is improved at the level of the IR in podocytes, either through IR over-expression or PTP1B knockdown, podocytes are protected from the development of ER stress. However, knockdown of PTEN, which improves insulin sensitivity through the PI3K-Akt pathway downstream of insulin, potentiates ER stress. Inhibition of mitogen-activated protein kinase kinase (MEK) or extracellular signal-regulated kinase (ERK) protects podocytes from ER stress. (IRS, insulin receptor substrate).

Defective insulin signalling in podocytes is associated with the development of ER stress^11^, and we hypothesised that by improving insulin sensitivity, podocytes would be protected from ER stress. We tested this in three genetic models of improved insulin sensitivity. Here we demonstrate that IR over-expression protects podocytes from ER stress and apoptosis, induced by either palmitate or by culturing in a diabetic environment rich in glucose, insulin and inflammatory cytokines, TNFα and IL-6 (Fig. 6).

PTP1B knockdown, which also improved insulin sensitivity, also protected podocytes from the development of ER stress (Fig. 6). Since PTP1B attenuates insulin signalling by dephosphorylating IR and IRS1, both IR over-expression and PTP1B knockdown act to improve insulin sensitivity at the level of the IR. However, it is clear that PTP1B has a wide range of roles, not all related to insulin signalling. Studies in other cell types suggest that PTP1B could be an active player in the UPR; for example PTP1B knock-out from mouse embryonic fibroblasts (MEFs) impaired IRE1-induced ER stress^27^. PTP1B is resident at the ER, an ideal site for its involvement in the UPR; it dephosphorylates the IR at membrane contact sites, either ER-plasma membrane sites or ER-endosomal sites when the IR is internalised^28,29^. Furthermore, PTP1B deficiency in the liver protects against high fat-induced ER stress^30,31^. This correlates with our observation, that PTP1B knockdown protects podocytes from ER stress, but other, apparently unrelated PTP1B activities, such as its role in the downregulation of VEGF signalling^32^, make the possibility of PTP1B as a good therapeutic drug target for the prevention of ER stress, podocyte injury and the development of DN more complicated.

PTEN was originally identified as a tumour suppressor protein and has been found to be deleted or mutated in many cancers^33^. It is a negative regulator of the PI3K-Akt pathway and becomes upregulated in podocytes derived from insulin receptor substrate 2 (IRS2)-knockout mice (*Irs2*^−/−^) to cause insulin resistance^34^; likewise, we demonstrate here that PTEN knockdown increases insulin signalling. However, unexpectedly, PTEN knockdown actually increased the development of ER stress rather than reducing it (Fig. 6). One of the many cellular effects of Akt signalling is a general increase in protein translation level, increasing protein load in the ER and making the development of ER stress more likely as the cell struggles to cope. Following this, PTEN might operate to reduce protein load by tempering Akt activity. Studies in PTEN^+/−^ and PTEN^−/−^ MEFs, which used PTEN knockdown as a means to increase Akt activity, demonstrated that knockdown of ectonucleoside triphosphate diphosphohydrolase 5 (ENTPD5), which hydrolyzes UDP in the ER to promote protein *N*-glycosylation and refolding, increased CHOP and GRP78 expression while at the same time reducing protein glycosylation^35,36^. Similarly, in primary cultures of PTEN^+/−^ and PTEN^−/−^ chrondrocytes, hypoxic conditions increased BiP/GRP78 expression, and this was negatively correlated with PTEN expression, with homozygous PTEN^−/−^ chrondrocytes displaying increased BiP expression compared with heterozygous PTEN^+/−^ chrondrocytes^37^. Our high throughput CHOP assay has enabled us to understand the relationship between ER stressor concentration and ER stress response in more detail, and we found particularly that CHOP expression was increased in untreated PTEN kd podocytes, and across multiple palmitate concentrations. Only for the highest concentration of palmitate, 10^−3^ M, and for differentiation of podocytes in diabetic media, did PTEN kd not further increase CHOP expression, potentially because CHOP expression is maximally induced under these conditions.

Our findings clearly indicate that the protection from ER stress bestowed on podocytes from improving insulin sensitivity is not straightforward. We show that improving insulin sensitivity at the level of the insulin receptor protects against ER stress. However, by genetically suppressing a downstream element of the insulin signalling network, PTEN, ER stress is enhanced. This indicates that there are critical nodes in the network that need to be dynamically controlled. In our genetic model we switched on the PI3K pathway constantly preventing upstream pathway modulation. A number of key pathways in the podocyte have been elegantly shown to be dependent on dynamic control, with either too much or too little activity being detrimental. These include VEGF-A^38^, mammalian target of rapamycin (mTOR)^39^, and mTORC1^40^. Insulin signals through the Raf-MEK-ERK pathway as well as the PI3K-Akt pathway in podocytes^8^, and much cross-talk is known to occur between the two pathways^41^. To investigate the mechanism by which PTEN knockdown sensitised podocytes to ER stress, we treated PTEN kd cells with MEK and ERK inhibitors and found surprisingly that inhibition of the MEK/ERK pathway suppressed the aberrant upregulation of CHOP seen in PTEN kd cells. Palmitate has been shown to have a number of molecular targets in different cell types; it is understood to disrupt calcium handling^42^, as well as activate mTORC1^43^ and FOXO1^44^. Future work will focus on the interplay between the PI3K-Akt and Raf-MEK-ERK signalling pathways and how their dynamic regulation can influence the ER stress response.

Here we report the development of a robust antibody-based high content imaging assay for ER stress in podocytes. This assay monitors the increase in nuclear CHOP fluorescence, which is quantifiable when cells are segmented using a DAPI (or other nuclear) stain, using simple automated algorithms. Immortalised podocytes are grown in black-walled 96-well plates optimised for imaging, making this assay amenable to the assessment of novel compounds to prevent ER stress in drug screens, potentially even in other cell types grown in culture. Furthermore, this assay enabled us to monitor the ER stress response in single cells rather than in the whole cell population, used here to consider the contribution of the quantity of PTP1B present in each podocyte, and the positive correlation between this measure and ER stress. To develop the CHOP assay we first screened more than 25 commercial antibodies specific for components of the UPR for their ability to recognise an expected change in protein fluorescence localisation or intensity. Only one other assay, the Herp assay, responded to thapsigargin and tunicamycin as would be predicted, by upregulating Herp expression in the cytoplasm (Supplementary Fig. 2). However, Herp was not observed to be upregulated in response to the endogenous ER stressor, palmitate, and so this assay was not taken any further.

In conclusion, we have developed a robust high content imaging assay for ER stress in podocytes, which we have used alongside established ER stress-specific luciferase assays to explore the relationship between insulin sensitivity and ER stress. We hypothesised that by improving insulin sensitivity, podocytes would be protected from ER stress, with the corollary that the reduced insulin sensitivity in type 2 diabetes would predispose podocytes to ER stress, increasing GFB permeability and promoting DN. Consistent with this working hypothesis, we found that improving insulin sensitivity at the level of the IR, by over-expression of a positive regulator (the IR itself) or knockdown of a negative regulator (PTP1B), protected podocytes from ER stress induced by palmitate or diabetic media. Conversely, we also found that knockdown of a negative regulator of the PI3K-Akt pathway, PTEN, actually increased ER stress despite also improving insulin sensitivity. Our study suggests that effectors other than Akt mediate the protective effect of insulin against ER stress, and also highlights the potential for enhancing insulin receptor activity to prevent podocyte injury and the development of DN.

## Methods

### Cell lines

Mouse podocytes immortalised by transduction with a temperature sensitive large T antigen SV40 construct as previously described^45,46^, were used as wt cells and as a background for making wt-IR, PTEN kd and PTP1B kd cell lines. The human IR sequence (NM_000208.2) in pLenti-TetCMV(IR)-Rsv(RFP-Bsd) expression vector (Gentarget) was co-transfected into Lenti-X 293 T cells (Clontech) with the packaging vectors pMD2.G (Addgene plasmid #12259) and psPAX2 (Addgene plasmid #12260) as previously reported^47^. Immortalised podocytes were transduced for 16 hr with the resulting lentivirus together with 8 µg/ml polybrene. IR-overexpressing podocytes were then selected using 15 µg/ml blasticidin. PTEN and PTP1B were stably knocked down using MISSION® shRNA lentiviral particles (PLKO-puro plasmid backbone, Sigma-Aldrich) and selected with 0.5 µg/ml puromycin. To produce the ER stress transcriptional reporter cell lines, immortalised cells from each genetic background (wt, wt-IR, PTEN kd, PTP1B kd) were transduced for 16 hr with lentiviral particles (MOI 50) from the Cignal Lenti Reporter Assay (QIAGEN), in the presence of 8 µg/mL polybrene. Transduced cells were selected with 0.5 µg/mL puromycin, where possible. Specifically, for each background, the following cell lines were created: cell lines expressing the promoter sequence regulated by the ATF6 transcription factor driving firefly luciferase expression (CLS-6031L, QIAGEN); the ERSE (NF-Y/CBF and YY1 binding elements) driving firefly luciferase expression (CLS-9032L, QIAGEN); or constitutively-expressed firefly luciferase positive control (CCS-PCL, QIAGEN). All transcriptional reporter lines additionally expressed renilla luciferase (CLS-RCL, QIAGEN) as an internal positive control.

### Cell culture and treatments

Immortalised cells are routinely cultured in RPMI-1640, 10% FCS and penicillin-streptomycin (Sigma-Aldrich) at 33 °C in a humidified atmosphere at 5% CO_2_. The day after plating for an experiment, the media was changed and cells thermoswitched to 37 °C for 10–14 days differentiation. For treatment with diabetic media, podocytes were cultured in RPMI-1640, 25 mM glucose (above that present in RPMI-1640), 100 nM insulin (human recombinant protein expressed in yeast, Bio Techne), 1 ng/ml TNFα (recombinant mouse N-terminal truncated form (aa 84–235), R & D Systems), 1 ng/ml IL-6 (recombinant mouse, R & D Systems)^25^ for the duration of the differentiation period, with regular changes of media. For treatment with thapsigargin (Sigma-Aldrich), tunicamycin (Tocris) or palmitate (the ER stressors), the media was removed 18 hr (luciferase assay) or 24 hr (high content imaging) before lysis or fixation respectively, and replaced with fresh media containing the appropriate concentration of ER stressor. For treatment with MEK (PD 184352, 10 µM, PZ0181-5MG, Sigma-Aldrich) or ERK (FR 180204, 30 µM, 5-(2-Phenyl-pyrazolo[1,5-a]pyridin-3-yl)-1H-pyrazolo[3,4-c]pyridazin-3-ylamine, #3706, Tocris) inhibitors, palmitate dilutions were prepared in diluted inhibitor and cells were treated alongside controls for 24 hr. Palmitate was prepared as described^48^; sodium palmitate and bovine serum albumin (fatty acid-free) were purchased from Sigma-Aldrich. For insulin stimulation, cells were serum-starved for 4 hr prior in 100 µl RPMI-1640, 0.1% FCS. Insulin concentration was prepared at 5 × concentrated and added in 25 µl to the existing media in the cell culture plate at the appropriate timepoint.

### Western blotting and antibodies

Protein expression was analysed from whole cell lysates as previously described^25^, using primary antibodies recognising GRP78 (BiP) (C50B12 #3177, Cell Signaling Technology (CST)), CHOP (DDIT3, ab11419, Abcam), PTEN (#9552, Cell Signaling Technology), PTP1B (N-terminal, SAB4502525, Sigma-Aldrich), insulin receptor β (4B8 #3025, Cell Signaling Technology), β-actin (#A1978 Sigma-Aldrich), and GAPDH (#E12-042, EnoGene), p-PERK (#3179, CST), total PERK (#3192, CST).

### Luciferase assays

Luciferase assays were performed using the Dual-luciferase® reporter assay (#E1980, Promega), following the manufacturer’s instructions. Cells seeded onto 96-well tissue culture-treated plates at 3 × 10^3^ cells/well, differentiated and treated were washed twice in PBS, lysed in 20 µl passive lysis buffer at room temperature with rocking, and frozen briefly at −20 °C. Each plate was then read consecutively with the luciferase and renilla-specific substrates on a MLX Luminometer (Dynex Technologies). The total relative light units (RLU) produced in each well for 6 s by the firefly luciferase reporters was normalised to the corresponding RLU measurement for the renilla luciferase control in the same lysates.

### High content imaging

Cells were seeded into Costar black-walled 96-well plates (Corning #3904, Arlington, UK) at 1–3 × 10^3^ cells/well in 100 µl normal growth medium. After stimulation, cells were fixed and analysed as described^49^. After blocking in 5% normal goat serum (NGS) (Sigma-Aldrich), the cells were incubated with appropriate primary antibody in 1% NGS, PBS at room temperature with rocking for 2 hr: (phospho-) p-Akt (S473) (D9E) XP(R) rabbit monoclonal antibody (#4060, Cell Signaling Technology) (1:200); CHOP (DDIT3, ab11419, Abcam) (1:200); PTP1B (ab189179, Abcam) (1:200); Herp (Herpud, ab150424, Abcam) (1:200). The cells were washed three times with 100 µl/well PBS, and then incubated with secondary antibody, Alexa Fluor 488 or 546 (Molecular probes) 1:200 in 1% NGS in PBS, at room temperature with rocking for 1 hr. The cells were washed twice and then incubated with DAPI, 300–600 nM in PBS, 100 µl per well, covered at room temperature with rocking for 20 min. Lastly, the cells were washed twice with PBS; the second wash remained on the cells for imaging.

Imaging in live cells was carried out for apoptosis. Following palmitate treatment, the cell culture media was tipped off and replaced with live cell imaging buffer^50^ containing 2 µM CellEvent Caspase 3/7 green fluorescent dye (C10723, Molecular Probes) and Hoechst 33342 dye (Molecular Probes). The cells were incubated in the dark at 37 °C for 30 min prior to imaging.

High content imaging was performed using the IN Cell Analyzer 1000 or 2200 system (GE Healthcare) with a 10 × objective and filters for DAPI (blue channel), Alexa488 (green channel) or Alexa647 (far red channel) (wt-IR cells express red fluorescent protein as a marker). Experiments were carried out with treatments in duplicate or triplicate wells. Three fields were imaged per well such that 300–1500 cells were typically imaged per well. Image analysis was performed using IN Cell Analyzer 1000 Workstation Multi-Target Analysis algorithms using DAPI staining to segment the nucleus. For PTP1B, raw nuclear intensity values in arbitrary fluorescence units (AFU) were used. For CHOP and CellEvent Caspase 3/7 measures, AFU was adjusted relative to vehicle control for wt podocytes (1:10,000 DMSO for Thapsigargin and Tunicamycin; 1% BSA for Palmitate) or wt podocytes grown in normal growth media (to control for diabetic media). For Herp, the fluorescence intensity in a 3 µm collar around the nucleus was plotted. For pAkt, frequency distribution plots for wt cells of nuclear fluorescence intensity values for unstimulated and highest insulin concentration were used to determine appropriate cut-offs for cells positive for pAkt in the nucleus. Unstimulated wt cells were deemed to have ~10% positive cells, and the same cut-off value was used for all cell types in a single experiment. Multiple experiments were combined by normalising the datasets to the lowest unstimulated values for each cell type.

For sorting of cells based on PTP1B expression, wt and PTP1B kd were untreated or treated with 300 µM palmitate for the final 24 hr of the differentiation period, 8 wells per condition. Cells were fixed and permeabilised, then co-stained with CHOP and PTP1B antibodies, followed by Alexa Fluor goat anti-mouse 488 or anti-rabbit 546, respectively, and DAPI. Four fields per well were imaged using the IN Cell Analyzer 2200 system (GE Healthcare). A perl script (available on request) was used to extract nuclear CHOP and nuclear PTP1B fluorescence intensity values for single cells. Perl software version: Strawberry Perl 5.20.2.1–64bit-portable. WT and PTP1B kd cells were combined *in silico* to yield a broad range of PTP1B values. Cells were sorted based on the amount of PTP1B expressed and placed into 50-AFU bins. Mean CHOP values within each bin were determined and standard deviation values calculated.

## Electronic supplementary material

Supplementary information

## References

[1] Marshall SM (2012). Diabetic nephropathy in type 1 diabetes: has the outlook improved since the 1980s?. Diabetologia.

[2] Rosolowsky ET (2011). Risk for ESRD in type 1 diabetes remains high despite renoprotection. J. Am. Soc. Nephrol..

[3] United States Renal Data System. 2013 Atlas of End-Stage Renal Disease. At https://www.usrds.org/2013/pdf/v2_ch1_13.pdf (2013).

[4] Andersen AR, Christiansen JS, Andersen JK, Kreiner S, Deckert T (1983). Diabetic nephropathy in Type 1 (insulin-dependent) diabetes: an epidemiological study. Diabetologia.

[5] Krolewski AS, Warram JH, Christlieb AR, Busick EJ, Kahn CR (1985). The changing natural history of nephropathy in type I diabetes. Am. J. Med..

[6] Wolf G, Chen S, Ziyadeh FN (2005). From the periphery of the glomerular capillary wall toward the center of disease: podocyte injury comes of age in diabetic nephropathy. Diabetes.

[7] Reddy GR, Kotlyarevska K, Ransom RF, Menon RK (2008). The podocyte and diabetes mellitus: is the podocyte the key to the origins of diabetic nephropathy?. Curr. Opin. Nephrol. Hypertens..

[8] Welsh GI (2010). Insulin signaling to the glomerular podocyte is critical for normal kidney function. Cell Metab..

[9] Cao Y (2014). Role of endoplasmic reticulum stress in apoptosis of differentiated mouse podocytes induced by high glucose. Int. J. Mol. Med..

[10] Tao J-L (2012). Endoplasmic reticulum stress is involved in podocyte apoptosis induced by saturated fatty acid palmitate. Chin. Med. J..

[11] Madhusudhan T (2015). Defective podocyte insulin signalling through p85-XBP1 promotes ATF6-dependent maladaptive ER-stress response in diabetic nephropathy. Nat. Commun..

[12] Zhuang A, Forbes JM (2014). Stress in the kidney is the road to pERdition: is endoplasmic reticulum stress a pathogenic mediator of diabetic nephropathy?. J. Endocrinol..

[13] Kim T-Y, Kim E, Yoon SK, Yoon J-B (2008). Herp enhances ER-associated protein degradation by recruiting ubiquilins. Biochem. Biophys. Res. Commun..

[14] Back SH, Kaufman RJ (2012). Endoplasmic reticulum stress and type 2 diabetes. Annu. Rev. Biochem..

[15] Zinszner H (1998). CHOP is implicated in programmed cell death in response to impaired function of the endoplasmic reticulum. Genes Dev..

[16] Oyadomari S, Mori M (2004). Roles of CHOP/GADD153 in endoplasmic reticulum stress. Cell Death Differ..

[17] Gotoh T, Terada K, Oyadomari S, Mori M (2004). hsp70-DnaJ chaperone pair prevents nitric oxide- and CHOP-induced apoptosis by inhibiting translocation of Bax to mitochondria. Cell Death Differ..

[18] Chen Y (2014). Down-regulation of PERK-ATF4-CHOP pathway by Astragaloside IV is associated with the inhibition of endoplasmic reticulum stress-induced podocyte apoptosis in diabetic rats. Cell. Physiol. Biochem..

[19] Coward RJM (2005). The human glomerular podocyte is a novel target for insulin action. Diabetes.

[20] Tejada T (2008). Failure to phosphorylate AKT in podocytes from mice with early diabetic nephropathy promotes cell death. Kidney Int..

[21] Hagenfeldt L, Wahren J, Pernow B, Räf L (1972). Uptake of individual free fatty acids by skeletal muscle and liver in man. J. Clin. Invest..

[22] Miles JM (2003). Nocturnal and postprandial free fatty acid kinetics in normal and type 2 diabetic subjects: effects of insulin sensitization therapy. Diabetes.

[23] Lennon R (2009). Saturated fatty acids induce insulin resistance in human podocytes: implications for diabetic nephropathy. Nephrol. Dial. Transplant.

[24] Sieber J (2010). Regulation of podocyte survival and endoplasmic reticulum stress by fatty acids. AJP: Renal Physiology.

[25] Lay, A. C. *et al* Prolonged exposure of podocytes to insulin induces insulin resistance through lysosomal and proteasomal degradation of the insulin receptor. *Diabetologia* (2017). 10.1007/s00125-017-4394-0 PMC6448913 28852804

[26] Galic S (2005). Coordinated regulation of insulin signaling by the protein tyrosine phosphatases PTP1B and TCPTP. Mol. Cell. Biol..

[27] Gu F (2004). Protein-tyrosine phosphatase 1B potentiates IRE1 signaling during endoplasmic reticulum stress. J. Biol. Chem..

[28] Stuible M, Tremblay ML (2010). In control at the ER: PTP1B and the down-regulation of RTKs by dephosphorylation and endocytosis. Trends Cell Biol..

[29] Popov D (2012). Endoplasmic reticulum stress and the on site function of resident PTP1B. Biochem. Biophys. Res. Commun..

[30] Delibegovic M (2009). Liver-specific deletion of protein-tyrosine phosphatase 1B (PTP1B) improves metabolic syndrome and attenuates diet-induced endoplasmic reticulum stress. Diabetes.

[31] Bence KK (2010). Hepatic PTP1B Deficiency: The Promise of a Treatment for Metabolic Syndrome?. J. Clin. Metab. Diabetes.

[32] Nakamura Y (2008). Role of protein tyrosine phosphatase 1B in vascular endothelial growth factor signaling and cell-cell adhesions in endothelial cells. Circ. Res..

[33] Salmena L, Carracedo A, Pandolfi PP (2008). Tenets of PTEN tumor suppression. Cell.

[34] Santamaria B (2015). IRS2 and PTEN are key molecules in controlling insulin sensitivity in podocytes. Biochim. Biophys. Acta.

[35] Fang M (2010). The ER UDPase ENTPD5 promotes protein N-glycosylation, the Warburg effect, and proliferation in the PTEN pathway. Cell.

[36] Shen Z, Huang S, Fang M, Wang X (2011). ENTPD5, an endoplasmic reticulum UDPase, alleviates ER stress induced by protein overloading in AKT-activated cancer cells. Cold Spring Harb. Symp. Quant. Biol..

[37] Yang G (2008). PTEN deficiency causes dyschondroplasia in mice by enhanced hypoxia-inducible factor 1alpha signaling and endoplasmic reticulum stress. Development.

[38] Eremina V (2003). Glomerular-specific alterations of VEGF-A expression lead to distinct congenital and acquired renal diseases. J. Clin. Invest..

[39] Gödel M (2011). Role of mTOR in podocyte function and diabetic nephropathy in humans and mice. J. Clin. Invest..

[40] Inoki K (2011). mTORC1 activation in podocytes is a critical step in the development of diabetic nephropathy in mice. J. Clin. Invest..

[41] Rozengurt E, Soares HP, Sinnet-Smith J (2014). Suppression of feedback loops mediated by PI3K/mTOR induces multiple overactivation of compensatory pathways: an unintended consequence leading to drug resistance. Mol. Cancer Ther..

[42] Ly LD (2017). Oxidative stress and calcium dysregulation by palmitate in type 2 diabetes. Exp. Mol. Med..

[43] Yasuda M (2014). Fatty acids are novel nutrient factors to regulate mTORC1 lysosomal localization and apoptosis in podocytes. Biochim. Biophys. Acta.

[44] Martinez SC (2008). Inhibition of Foxo1 protects pancreatic islet beta-cells against fatty acid and endoplasmic reticulum stress-induced apoptosis. Diabetes.

[45] Saleem MA (2002). A Conditionally Immortalized Human Podocyte Cell Line Demonstrating Nephrin and Podocin Expression. J. Am. Soc. Nephrol..

[46] Keir LS (2015). Generating conditionally immortalised podocyte cell lines from wild-type mice. Nephron.

[47] Rollason R (2016). Disease causing mutations in inverted formin 2 regulate its binding to G-actin, F-actin capping protein (CapZ α-1) and profilin 2. Biosci. Rep..

[48] Xu S (2015). Palmitate induces ER calcium depletion and apoptosis in mouse podocytes subsequent to mitochondrial oxidative stress. Cell Death Dis..

[49] Garner KL (2016). Information Transfer in Gonadotropin-releasing Hormone (GnRH) Signaling: Extracellular Signal-Regulated Kinase (Erk)-Mediated Feedback Loops Control Hormone Sensing. J. Biol. Chem..

[50] Garner KL (2017). Information Transfer via Gonadotropin-Releasing Hormone Receptors to ERK and NFAT: Sensing GnRH and Sensing Dynamics. J. Endocr. Soc..

