# Supplementary material for: Enhanced insulin receptor, but not PI3K, signalling protects podocytes from ER stress
Source: Sci Rep. 2018 Mar 2;8:3902. doi: 10.1038/s41598-018-22233-9 (PMC5834602; doi:10.1038/s41598-018-22233-9)

Enhanced insulin receptor, but not PI3K, signalling protects podocytes from ER stress

Kathryn L Garner, Virginie M S Betin, Vanda Pinto, Mark Graham,
Emmanuelle Abgueguen, Matt Barnes, David C Bedford, Craig A McArdle, Richard J M Coward.

Supplementary Figure 1: Treatment of wild-type podocytes for 2 days or more promotes widespread cell loss. Podocytes were treated for 1, 2, 3 or 4 days with (a) 10^{-9}-10^{-6} M Thapsigargin, (b) 10^{-9}-10^{-5} M Tunicamycin, or (c) 10^{-5}-10^{-3} M Palmitate, before being fixed, stained with DAPI and imaged. Raw cell counts are shown for 3 fields per well, duplicate wells per condition.

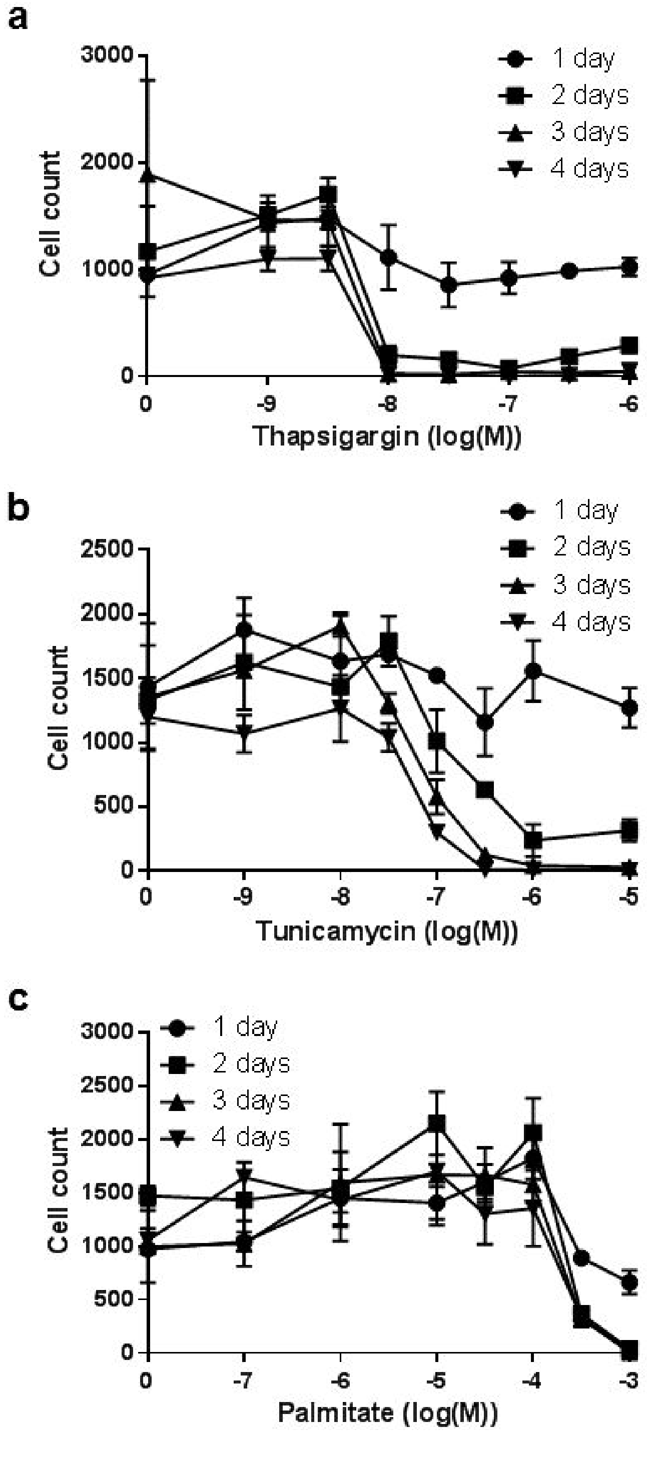

Supplementary Figure 2: GRP78 and p-PERK expression in wt and wt-IR podocytes treated with diabetic media. (a) WT or wt-IR podocytes cultured in normal growth media ('ctrl') or diabetic media ('D') during differentiation were treated without or with 300 μM palmitate for the final 24 hr before being lysed and subjected to western blotting for GRP78 (*n*=3). GRP78 expression normalised relative to β-actin loading control, then a ratio of palmitate-treated GRP78 divided by untreated GRP78 in each condition plotted. Paired *t* test, wt and wt + D * $p<0.05$, wt-IR and wt-IR + D, not significant ('ns'). Unpaired *t* test with Welch's correction, wt + D and wt-IR + D ** $p<0.01$. (b) The same samples as in (a) were additionally subjected to western blotting for phospho (p)-PERK, normalised relative to total PERK as a loading control (*n*=3). Paired *t* test, wt and wt + D *** $p<0.001$, wt-IR and wt-IR + D, not significant ('ns'). Unpaired *t* test with Welch's correction, wt + D and wt-IR + D * $p<0.05$.

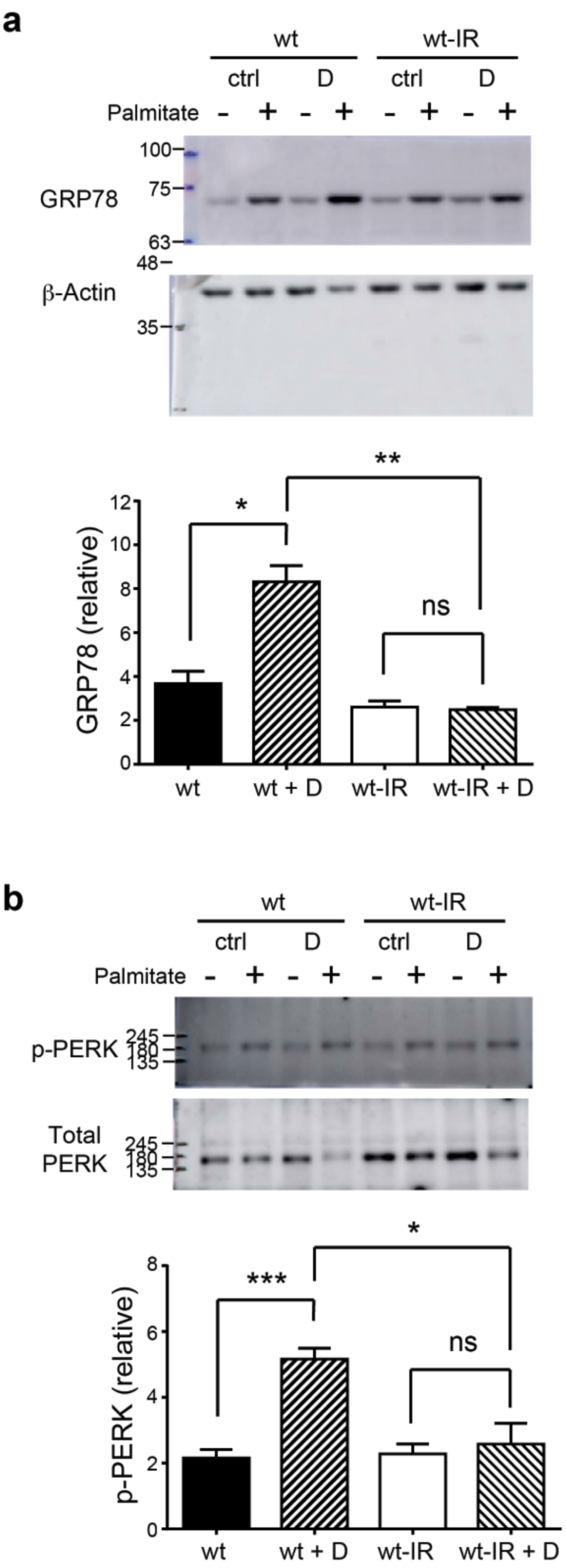
a
wt
wt-IR
ctrl
D
ctrl
D
Palmitate - + - + - + - +
GRP78
β-Actin
100
75
63
48
35
GRP78 (relative)
*
**
ns
0
2
4
6
8
10
12
wt
wt + D
wt-IR
wt-IR + D
b
wt
wt-IR
ctrl
D
ctrl
D
Palmitate - + - + - + - +
p-PERK
Total PERK
245
180
135
p-PERK (relative)
***
*
ns
0
2
4
6
8
wt
wt + D
wt-IR
wt-IR + D

Supplementary Figure 3: Herp assay for ER stress in podocytes. Podocytes treated with 10^{-9}-10^{-6} M Thapsigargin, 10^{-9}-10^{-5} M Tunicamycin or 10^{-5}-10^{-3} M Palmitate for 24 hr were fixed and permeabilised, stained with DAPI and immunostained for Herp. Cells were imaged and cytoplasmic intensity of Herp staining was quantified in a 3 µm collar around the nucleus.

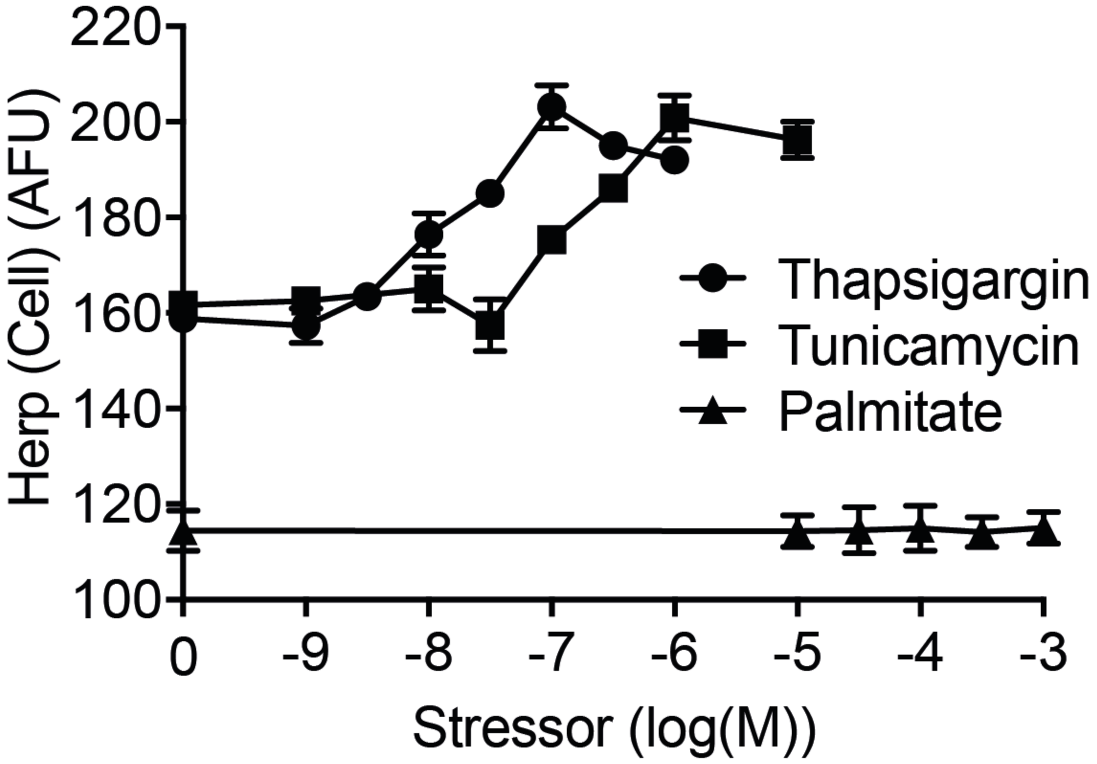

Supplementary Figure 4: GRP78, CHOP, IR, PTEN and PTP1B expression by western blot. (a) GRP78 expression in wt podocytes untreated or treated with 300 μM palmitate for 24 hr. (Cropped version shown in Fig. 1 c.) (b) CHOP expression in wt podocytes untreated or treated for 24 hr with 100 nM thapsigargin (‘TG’), 1 μM tunicamycin (‘Tuni’), or 300 μM palmitate (‘PA’). (Cropped version shown in Fig. 1 f.) (c) IRβ subunit and β-actin expression in wt-IR podocytes compared with wt cells. (Cropped version shown in Fig. 2 a.) (d) PTP1B and GAPDH expression in PTP1B kd cells compared with wt and cells transduced with a lentivirus containing a scrambled (scr) sequence. (Cropped version shown in Fig. 3 a.) (e) PTEN and β-actin expression in PTEN kd cells compared with wt and scr cells. Three repeat experiments shown. (Cropped version shown in Fig. 5 a.)

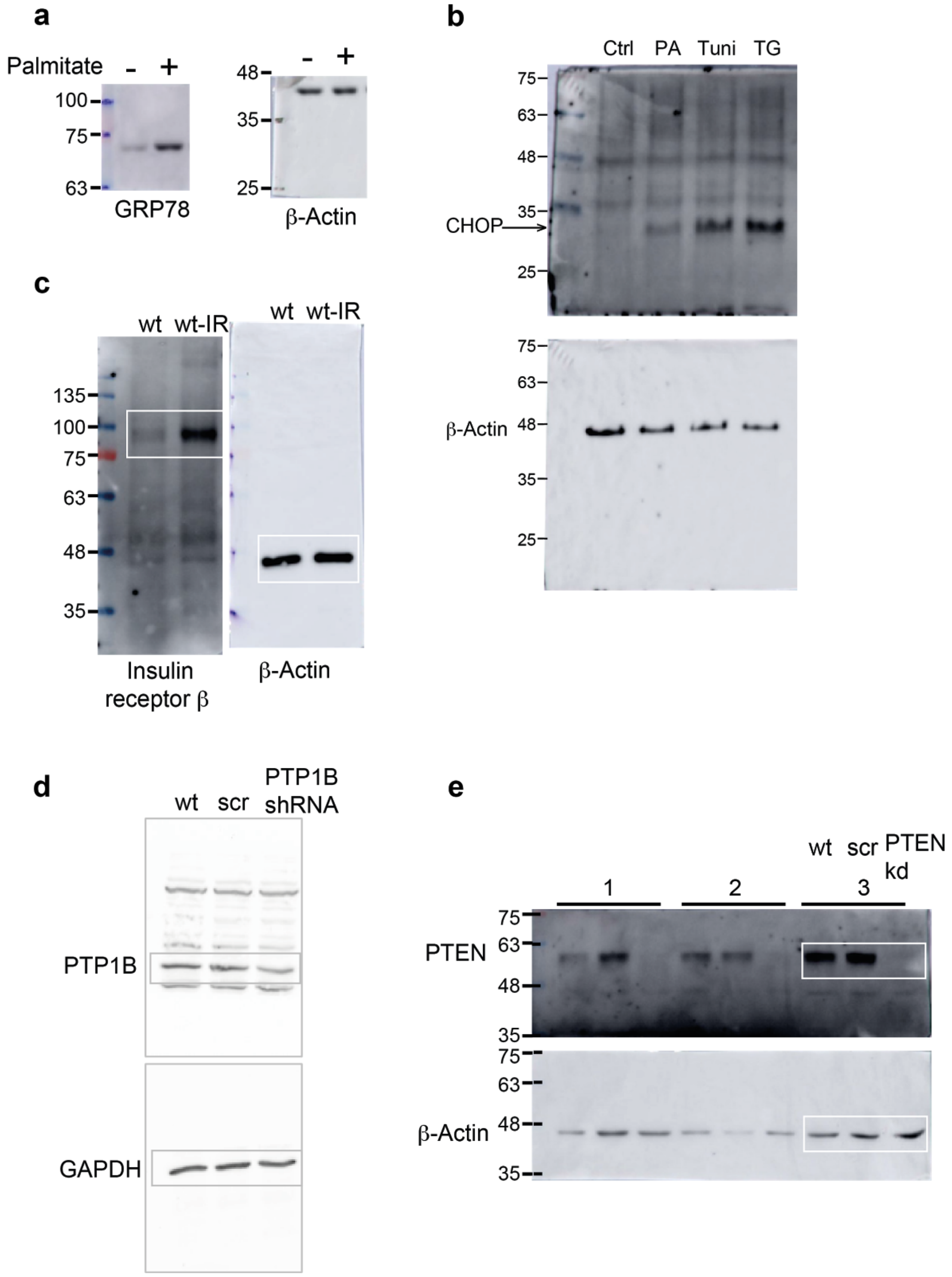

Supplement: Supplementary file 1 — Supplementary information [file 41598_2018_22233_MOESM1_ESM.pdf]
